# Circular RNA SMEK1 promotes neuropathic pain in rats through targeting microRNA-216a-5p to mediate Thioredoxin Interacting Protein (TXNIP) expression

**DOI:** 10.1080/21655979.2021.1965811

**Published:** 2021-09-14

**Authors:** Yufu Xin, Xinrong Song, Qingye Ge

**Affiliations:** 1 Department of Rehabilitation Medicine, The First Affiliated Hospital of Henan University of Science and Technology, Luoyang City, Henan Province, 471000, China; 2 Department of Rehabilitation Medicine of Chinese Medicine Hospital of PuYang Henan, Puyang City, Henan Province, 457000, China

**Keywords:** CircSMEK1, MicroRNA-216a-5p, microglia, neuropathic pain, inflammation

## Abstract

Neuropathic pain (NP) is a disease induced by damage to the nervous system. A large number of studies have manifested that circular RNAs (circRNAs) are key in the development of neurological diseases. However, the role of circRNA in NP remains ambiguous. In this study, the biological function and molecular mechanism of circSMEK1 were investigated in NP. NP rat and cell models were established by chronic contractile injury (CCI) surgery and lipopolysaccharide (LPS) treatment, separately. The results exposed that circSMEK1 and TXNIP were up-regulated in NP, while miR-216a-5p was down-regulated. The claw retraction threshold and claw retraction latency in rats were elevated and reduced separately via knockdown circSMEK1 and miR-216a-5p. Meanwhile, knockout circSMEK1 or elevated miR-216a-5p declined inflammatory cytokines tumor necrosis factor-α (TNF-α), interleukin (IL)-1β and IL6 in spinal cord, and the activation of microglia, but promoted the polarization of microglia into anti-inflammatory type, while up-regulation of circSMEK1 or knockdown of miR-216a-5p was opposite. Mechanism studies demonstrated that circSMEK1 mediated TXNIP expression through competitive adsorption of miR-216a-5p. Functional rescue experiments manifested that the suppressive effect of circSMEK1 knockdown on NP was reversed by declined miR-216a-5p simultaneously. In conclusion, the results of this study affirmed that circSMEK1 facilitates NP inflammation and microglia M1 polarization by modulating miR-216a-5p/TXNIP axis, providing a new molecular target for the future treatment of NP.

## 1. Introduction

Neuropathic pain (NP) is a type of intermittent or continuous burning pain, a pain syndrome featuring allodynia and hyperalgesia [1,2]. NP has become a serious health problem worldwide. It is reported that about 6.9–10% of the world’s population is affected by NP, and has paid a huge socio-economic cost for this [3]. The main causes of NP include neurological damage resulting from trauma and various diseases [4,5]. There have been various drugs for NP, like serotonin modulators, apoptosis inhibitors, etc., but their effects are still limited [6]. Now the molecular mechanism of NP is not fully clear yet, so it is very essential to understand it so as to find new curative targets.

Circular RNA (CircRNA), a long-chain non-coding RNA, is produced by reverse splicing of peptides with 5ʹ-3ʹ polarity or polyadenylate tail, featuring covalently closed loops [7]. A growing body of research suggests that circRNA is vital in neurological diseases, including cerebral ischemia and reperfusion [8], Alzheimer’s disease [9], meningitis [10] and sciatic nerve injury [11], etc. Recently, many studies have revealed the vital role of circRNAs in NP. Zhang Y *et al*. found that Circ_0005075, which targets miR-151a-3p, promotes NP in rats with chronic contractile injury (CCI) via inducing NOTCH2 expression [12]. Wei M *et al*. found that down-regulated circRNA zRANB1 mediates Wnt5a/β-Catenin signaling in CCI rat model, thus facilitating neuropathic pain through the miR-24-3p/LPAR3 axis 13. Additionally, a study reported that 188 circRNAs, 134 long non-coding RNAs, 12 miRNAs and 1,066 mRNAs were differentially expressed in the spinal cord after nerve injury [13]. CircSEMK1 is an important member of circRNA family. Based on previous studies, circSEMK1 is highly expressed in CCI rats [14]. But the underlying molecular mechanism by which circSEMK1 affects NP is still not clear.

This study was to explore the biological function of circSMEK1 in NP and its potential molecular mechanism. To achieve this goal, *in vivo* and *in vitro* models of NP were established, and circSMEK1 and miR-216a-5p expression was altered by transfection or lentivirus injection, assuring that circSMEK1 mediates Thioredoxin Interacting Protein (TXNIP) via adsorbing miR-216a-5p, thereby promoting NP inflammation and microglia M1 polarization.

## 2. Methods

### 2.1. Establishment and grouping of NP model

Adult male SD rats (200–250 g; 6 weeks old) from The First Affiliated Hospital of Henan University of Science and Technology Experimental Animal Center, were housed at 24 ± 2°C in a humidity of 50%-60% and a light-shade alternating cycle for 12 hours. The animals had free access to water and food. After one-week adaptive feeding, 84 rats were by random allocated into Sham group (n = 12) and CCI group (n = 72), via CCI to induce NP in rats [15]. In short, the rats were received anesthetization by intraperitoneal injection of sodium pentobarbital (40 mg/kg). Blunt anatomy shown the sciatic nerves on both sides, in isolation from surrounding tissues. The 4–0 catgut was employed to loosely ligate the sciatic nerve about 1 mm between ligatures. Rats in Sham group did not received ligation at the sciatic nerve. After operation, the muscle and skin layers were received with suture with thread, and the surgical site was treated with disinfection with iodine.

After CCI surgery, Hamilton syringe with a 30-gauge needle was inserted into the spinal subarachnoid between the L4 and L5 lumbar. After intrathecal injection of 10 μL of 2% lidocaine, the correct position of the catheter was received with verification via hind limb paralysis. Different recombinant lentiviruses (1 × 10^7^/0.1 ml) were given to rats by microneedle-based intrathecal injection for lentivirus infection. The lentiviral vectors of LV-NC, LV-circSMEK1, and LV-miR-216a-5p were received with synthetization in Shanghai Gene Pharmaceutical Co., Ltd. The rats were given sacrifice by cervical decapitation 14 days after lentivirus injection, and the spinal cord tissues of L4-L5 lumbar were collected. Some of them was received with fixation with 4% paraformaldehyde, and the rest were quickly stored at −80°C. This study was conducted in reference to the guidelines for the care and use of laboratory animals from the International Association for the Study of Pain, NIH. This work has got approval from the Animal Care and Use Committee of First Affiliated Hospital of Henan University of Science and Technology.

### 2.2. Pain threshold detection

Behavioral pain indicators were received with detection after lentivirus infection. Paw withdrawal threshold (PWT/g) and paw withdrawal latency (PWL/s) of each group were received with detection on 0, 1, 3, 7 and 14 days. PWT evaluation procedures were as follows: von Frey silk (Stoelting Co., Ltd., Wood Dale, IL, USA) was applied for stimulating rat paw to evaluate mechanical hypersensitivity. The instruments were placed in a quiet room, and the rats in a transparent organic glass cages (30 cm × 30 cm × 30 cm), the bottom of which was metal mesh fixed in a shelf of approximately 50 cm height. After adaption for 15 min, the test was started. With 0.008 g feng – fry silk to stimulate the frame feet of rat hind paws, the size was bigger and bigger. After stimulation, the acupuncture intensity (g) was recorded when the rats’ claws withdrew. Each thread was applied for stimulating rat paws for 5 times, with each time of stimulation in the whole process less than 1 min. Next, when the rat was placed in an organic glass box with radiation heat source, PWL measurement was conducted. The radiation heat source was concentrated on the footplate surface of the hind paws of rats. When the rats showed pain on the footplate and retracted their claws, the PWL was recorded [16].

### 2.3. Immunohistochemistry

Via microtome to cut the spinal cord tissue into 4-micron serial sections in thickness, after deparaffinization and rehydration, the sections were received with warm incubation with citrate buffer for antigen retrieval. Immunohistochemistry was performed as previously described [17]. The sections were received with block with 2% bovine serum albumin, and later with anti-Iba1 primary antibody (ab5076, Abcam) overnight at 4°C, after which the sections were given incubation with HRP-labeled secondary antibody at 37°C for 30 minutes. Later, the sections were received with staining with Diaminobenzidine working solution (Solarbio Life Sciences) in the dark for 5 minutes, via microscope to analyze protein expression.

### 2.4. Flow cytometry

Single-cell suspensions were obtained as same as before [18]. The spinal cord was removed from the spinal cord tissue by inflating, and the tissue was gently ground into single-cell suspension by using the plunger of a syringe through a 45-micron nylon net. Then the dissociation was performed in cells by Percoll (Amersham Pharmacia Biotech, Piscataway, NJ, USA) gradient centrifugation. Different fluorescent labeled antibodies were implemented to identify different immune cell subtypes (CD68, M0876, Dako; CD206, MCA2235, Bio-Rad). After incubation at 4°C for 30 min, the cells were washed three times with 0.01 M PBS (pH7.4), fixed with 1% PFA, and detected via BD Accuri flow cytometry (Becton Dickinson, San Diego, CA). Finally, the cell subsets were analyzed via FlowJo 7.6.1 software (TreeStar Inc., Ashland OR).

### 2.5. Cell culture

Rat microglia cell line HAPI from BeNa Culture Collection (China) was received with culture in Gibco high-glucose Dulbecco’s Modified Eagle Medium supplemented with 10% FBS and 5% CO_2_ at 37°C. Later, 100 ng/mL lipopolysaccharide (LPS; Sigma-Aldrich) was applied to stimulate the cells.

### 2.6. Cell transfection

Small interfering RNA targeting circSMEK1 (si-circSMEK1), negative control of small interfering RNA (si-NC), pcDNA3.1, pcDNA3.1-circSMEK1 overexpression vectors, mimic-NC and miR-216a-5p-mimic were from Shanghai Gene Pharmaceutical Co., Ltd. The microglia were placed in 6-well plates (2 × 10^6^/well). After reaching 70–80% confluence, Invitrogen Lipofectamine® 2000 from Thermo Fisher Scientific, Inc. was employed to transfect the above vectors or oligonucleotides into microglia in reference to the instructions [19]. After 48-hour transfection, cell collection was done for subsequent experiments.

### 2.7. Enzyme-linked immunosorbent assay (ELISA)

Seven days after the lentivirus injection, 3 rats in each group by random selected were sacrificed 7 days after the operation to collect spinal cord tissues. TNF-α, IL-6 and IL-1β levels in spinal cord were given detection based on the instructions of kits from Nanjing Jiancheng Institute of Biological Engineering (China).

### 2.8. Reverse transcription quantitative polymerase chain reaction (RT-qPCR)

RT-qPCR was conducted as mentioned before [20]. Total RNA extraction was done via Invitrogen TRIzol reagent (U.S.). Reverse transcription of total RNA was done via M-MLV reverse transcriptase (China) for mRNA detection, and TaqMan miRNA reverse transcription kit (Applied Biosystems, U.S.) was employed for miRNA detection. SYBR Green PCR Master Mix (Applied Biosystems) was applied for cDNA amplification, GAPDH and U6 as reference genes for mRNA and miRNA expression, respectively. Relative expression calculation was done via 2^−ΔΔCt^ method, primer sequences detailed in Table 1.

**Table 1.** RT-qPCR primer sequences

	Primer sequences (5’ – 3’)
GAPDH	Forward: 5ʹ- AATGGACAACTGGTCGTGGAC-3’
	Reverse: 5ʹ- CCCTCCAGGGGATCTGTTTG-3’
U6	Forward: 5ʹ- AGTAAGCCCTTGCTGTCAGTG-3’
	Reverse: 5ʹ- CCTGGGTCTGATAATGCTGGG-3’
MiR-216a-5p	Forward: 5ʹ- ACATCCTCGGCCAGTAAGACTG-3’
	Reverse: 5ʹ-GTCGACCAGATTGCGTTCG −3’
CircSMEK1	Forward: 5ʹ-CCTGGCAAAGATGGTGAGACAG-3’
	Reverse: 5ʹ- CCTGGTTTTCCACCTTCACCTG −3’

### 2.9. Western blot

Western blot was implemented accordingly [21]. RIPA Lysis Buffer (Sigma-Aldrich, U.S.) was applied to separate total protein from tissues and cells. Later, NanoDrop 2000 spectrophotometer (Thermo Scientific, U.S.) was employed to detect the total protein concentration. After loading 20 μg protein at the same amount, electrophoresis was done on 10% SDS-PAGE gel. Then, all proteins were transferred to PVDF membranes (Millipore Corp., U.S.). Later, the membranes were received with 1-hour incubation with 5% skimmed milk at room temperature, then with the following primary antibodies CD11b (11–0112, Invitrogen), CD68 (M0876, Dako), CD206 (MCA2235, Bio-Rad), TXNIP (ab188865, Abcam), β-actin (A5441, Millipore Sigma) to incubate overnight at 4°C. At last, all membranes were incubated with HRP-conjugated goat anti-rabbit or -mouse secondary antibody (Beijing TransGen Biotech Co., Ltd.). Protein band visualization was done via ECL kit (Applygen Technologies Inc.) combined with analysis via Image-Pro Plus 6.0 software.

### 2.10. Dual luciferase reporter (DLR) experiment

The potential binding sites of circSMEK1 or TXNIP and miR-216a-5p were replaced by circSMEK1 mutant (MUT) and TXNIP-3ʹUTR-MUT [22]. HEK297 cells were seeded in 96-well plates overnight. When the cells reached 50–70% confluence, circSMEK1-WT (or circSMEK1-MUT) or TXNIP-3ʹUTR-WT (or TXNIP-3ʹUTR-MUT) and miR-216a-5p mimic and mimic NC were given co-transfection into HEK-297 cells (BeNa Culture Collection, China). Forty-eight hours after transfection, the luciferase reporter activity measurement was done on Promega DLR system.

### 2.11. Data analysis

All data was detailed in mean ± standard deviation (SD). All statistical comparisons were done on SPSS version 19.0 (SPSS Inc., U.S.) via student t test and one-way analysis of variance (ANOVA). The difference was statistically significant (*P* < 0.05).

## 3. Results

### 3.1. Increased circSMEK1 expression in NP

**Figure 1.**
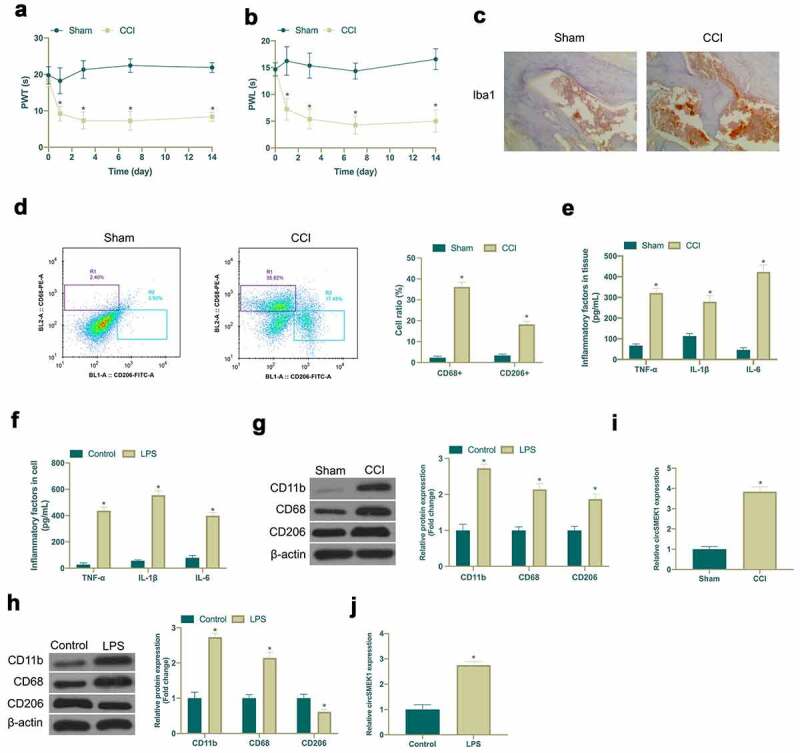
Elevated circSMEK1 expression in NP

Aiming to exploit the role of circSMEK1 in NP, a CCI rat model was established via surgery and LPS was employed to induce an *in vitro* NP model. After CCI surgery, CCI group showed obvious mechanical allodynia and thermal hyperalgesia, while Sham group did not have any abnormalities (Figure 1(a,b)). In addition, immunohistochemistry and flow cytometry were applied for detection of microglia activation marker Iba1 expression, as well as CD68+ (pro-inflammatory phenotype marker) and CD206+ (anti-inflammatory phenotype marker) proportion of cells. CCI surgery visually increased Iba1 positive cells and elevated the ratio of CD68+ and CD206+ cells in spinal cord (Figure 1(c,d)). Through ELISA examination, it was found that CCI surgery and LPS induction signally elevated TNF-α, IL-1β and IL-6 levels in spinal cord tissue and microglia (Figure 1(e,f)). CD11b (microglia marker), CD68 and CD206 expressions were checked via western blot. CD11b, CD68 and CD206 expression in spinal cord tissue and microglia was obviously increased via CCI surgery and LPS induction (Figure 1(g,h)). Subsequently, circSMEK1 expression detection was done by RT-qPCR. CircSMEK1 was elevated signally in spinal cord of CCI rats and LPS-induced microglia (Figure 1(i,j)). These findings indicated that NP *in vitro* and *in vivo* models have been successfully established, and circSMEK1 is highly expressed in NP.

A-B. Mechanical pain threshold and withdrawal latency to thermal stimulation in plantar test of Sham and CCI groups; C. Immunohistochemistry detection of Iba1 expression in spinal cord tissue of Sham and CCI groups; D. Flow cytometry detection of CD68+ and CD206+ cells in spinal cord tissues of Sham and CCI groups; E. ELISA detection of TNF-α, IL-1β and IL-6 levels in the spinal cord tissues of Sham and CCI groups; F. ELISA detection of TNF-α, IL-1β and IL-6 levels in microglia of Control and LPS groups; G. Western blot detection of CD11b, CD68 and CD206 expressions in spinal cord of Sham and CCI groups; H. Western blot detection of CD11b, CD68 and CD206 expression in microglia of Control and LPS groups; I. RT-qPCR detection of circSMEK1 expression in spinal cord tissue of Sham and CCI groups; J. RT-qPCR detection of circSMEK1 expression in microglia of Control and LPS groups; Data presentation was detailed as mean ± SD (A, B, D, F, H, n = 9; E, G, I, n = 3), * vs Sham or Control groups, *P* < 0.05.

### 3.2. CircSMEK1 knockdown improves NP, while overexpressed circSMEK1 aggravates it

**Figure 2.**
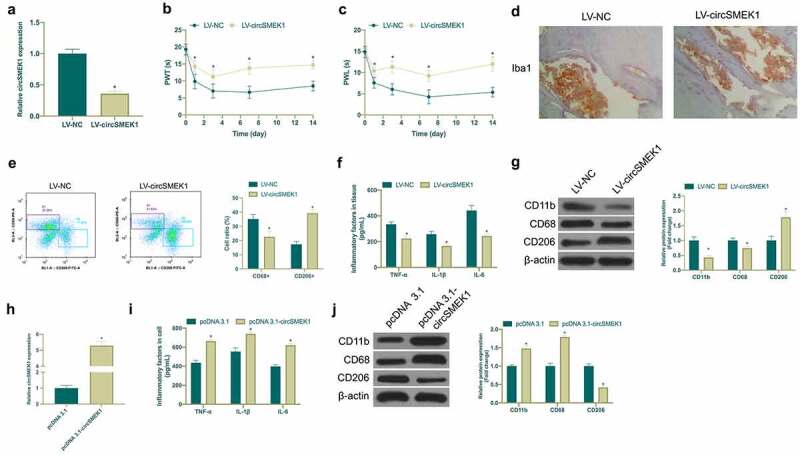
CircSMEK1 knockdown improves NP, while overexpressed circSMEK1 aggravates it

Next, the biological function of circSMEK1 was examined in NP. Then, through lentiviral injection, circSMEK1 was knocked down in CCI rats (Figure 2(a)), finding that mechanical allodynia and thermal hyperalgesia in rats were signally reduced (Figure 2(b,c)). Additionally, circSMEK1 knockdown apparently reduced Iba1 positive cells in spinal cord (Figure 2(d)). Flow cytometry manifested that knockdown circSMEK1 reduced the proportion of CD68+ cells and elevated the proportion of CD206 cells in the spinal cord (Figure 2(e)), and declined TNF-α, IL-1β and IL-6 levels (figure 2(f)). Pro-inflammatory/anti-inflammatory microglia phenotype test revealed that knocking down circSMEK1 reduced CD11b and CD68 expression but elevated CD206 expression (Figure 2(g)), indicating that this knockdown improves NP. Later, circSMEK1 was overexpressed in an *in vitro* model (Figure 2(h)), after which TNF-α, IL-1β and IL-6 levels in microglia were elevated visually (Figure 2(i)). Additionally, overexpressing circSMEK1 obviously increased CD11b and CD68 expression in LPS-induced microglia but inhibited CD206 expression (Figure 2(j)), indicating that such overexpression aggravates NP.

A. RT-qPCR detection of circSMEK1 expression in spinal cord tissue of LV-NC and LV-circSMEK1 groups; B-C. Mechanical pain threshold and heat-stimulated withdrawal latency in plantar test of LV-NC and LV-circSMEK1 groups; D. Immunohistochemistry detection of Iba1 expression in spinal cord of LV-NC and LV-circSMEK1 groups; E. Flow cytometry detection of CD68+ and CD206+ cells in spinal cord tissues of rats in LV-NC and LV-circSMEK1 groups; F. ELISA detection of TNF-α, IL-1β and IL-6 levels in spinal cord tissue of LV-NC and LV-circSMEK1 groups; G. Western blot detection of CD11b, CD68 and CD206 expressions in spinal cord tissue of LV-NC group and LV-circSMEK1 group; H. RT-qPCR detection of CD11b, CD68 and CD206 expressions in microglia of pcDNA3.1 and pcDNA3.1-CircSMEK1 groups; I. ELISA detection of TNF-α, IL-1β and IL-6 levels in microglia of pcDNA3.1 and pcDNA3.1-circSMEK1 groups; J. Western blot detection of CD11b, CD68 and CD206 expressions in microglia of pcDNA3.1 and pcDNA3.1-circSMEK1 groups; Data presentation detailed as mean ± SD (A-F, n = 9; G-I, n = 3); * vs LV-NC or pcDNA 3.1 groups, *P* < 0.05.

### 3.3. Knocking down miR-216a-5p promotes NP, whereas overexpressing miR-216a-5p inhibits it

**Figure 3.**
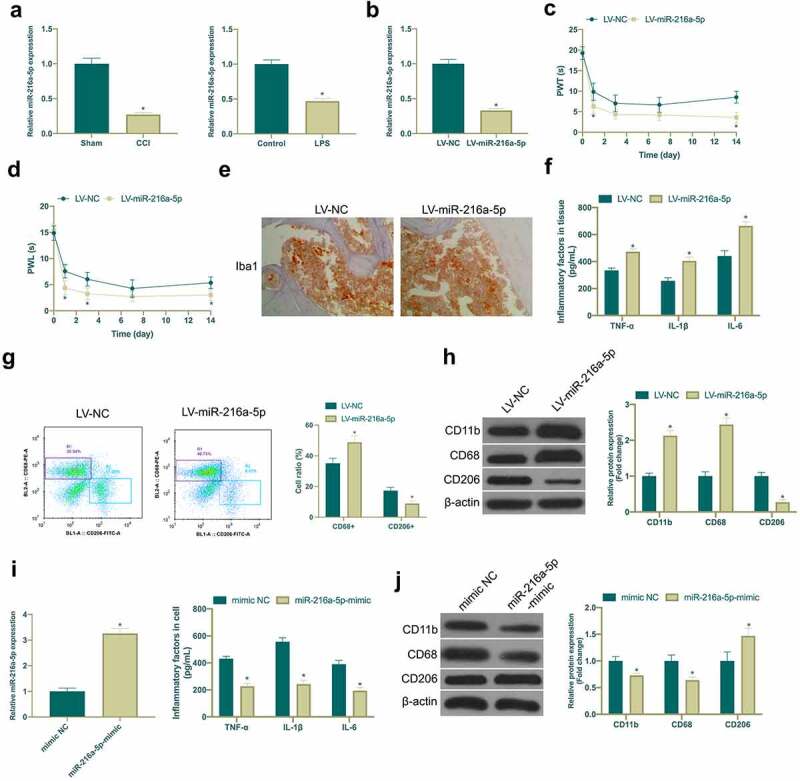
Knocking down miR-216a-5p promotes NP, but overexpressed miR-216a-5p represses it

MiR-216a-5p has been shown to be under-expressed in NP, but its mechanism in NP is still unclear [23]. In this study, it was found that miR-216a-5p was down-regulated in the spinal cord tissues of CCI rats and LPS-induced microglia cell model (Figure 3(a)). Subsequently, miR-216a-5p was silenced in CCI rats (Figure 3(b)), manifesting that after knockdown miR-216a-5p, mechanical abnormal pain and hypersensitivity to heat were aggravated in CCI rats (Figure 3(c,d)), and the number of Iba1 positive cells in spinal cord tissue was clearly elevated (Figure 3(e)), and there were elevated CD68+ cells and reduced CD206+ cells (figure 3(f)), and obviously up-regulated TNF-α, IL-1β and IL-6 (Figure 3(g)). Moreover, miR-216a-5p silence also strengthened CD11b and CD68 expression but curbs CD206 (Figure 3(h)). This indicated that miR-216a-5p was declined in neuropathic pain, and silenced miR-216a-5p promoted neuropathic pain. Subsequently, miR-216a-5p mimic was transfected into LPS-treated microglia cells for elevation of miR-216a-5p (Figure 3(i)), affirming that strengthening miR-216a-5p restrained TNF-α, IL-1β and IL-6 (Figure 3(j)), CD11b and CD68, and accelerated CD206 expression (Figure 3(k)), suggesting that up-regulated miR-216a-5p alleviated neuropathic pain.

A. RT-qPCR detection of miR-216a-5p expression in CCI rats and LPS-treated microglia; B. RT-qPCR detection of miR-216a-5p expression in spinal cord of LV-NC and LV-miR-216a-5p groups; C-D. Mechanical pain threshold and heat-stimulated withdrawal latency of thermal stimulation in plantar test of LV-NC and LV-miR-216a-5p groups; E. Immunohistochemistry detection of Iba1 expression in spinal cord of LV-NC and LV-miR-216a-5p groups; F. Flow cytometry detection of CD68+ and CD206+ cells in spinal cord tissues of rats in LV-NC and LV- miR-216a-5p groups; G. ELISA detection of TNF-α, IL-1β and IL-6 levels in spinal cord of LV-NC and LV-miR-216a-5p groups; H. Western blot detection of CD11b, CD68 and CD206 expressions in spinal cord tissue of LV-NC and LV-miR-216a-5p groups; I. RT-qPCR detection of circSMEK1 expression in microglia of mimic NC and miR-216a-5p-mimic groups; J. ELISA detection of TNF-α, IL-1β and IL-6 levels in microglia in mimic NC and miR-216a-5p-mimic groups; K. Western blot detection of CD11b, CD68 and CD206 expressions in microglia of mimic NC and miR-216a-5p-mimic groups; Data presentation detailed as mean ± SD (A, n = 9 or 3; B-G, n = 9; H-J, n = 3); * vs LV-NC or mimic NC groups, *P* < 0.05.

### 3.4. CircSMEK1 competitively binds to miR-216a-5p

**Figure 4.**
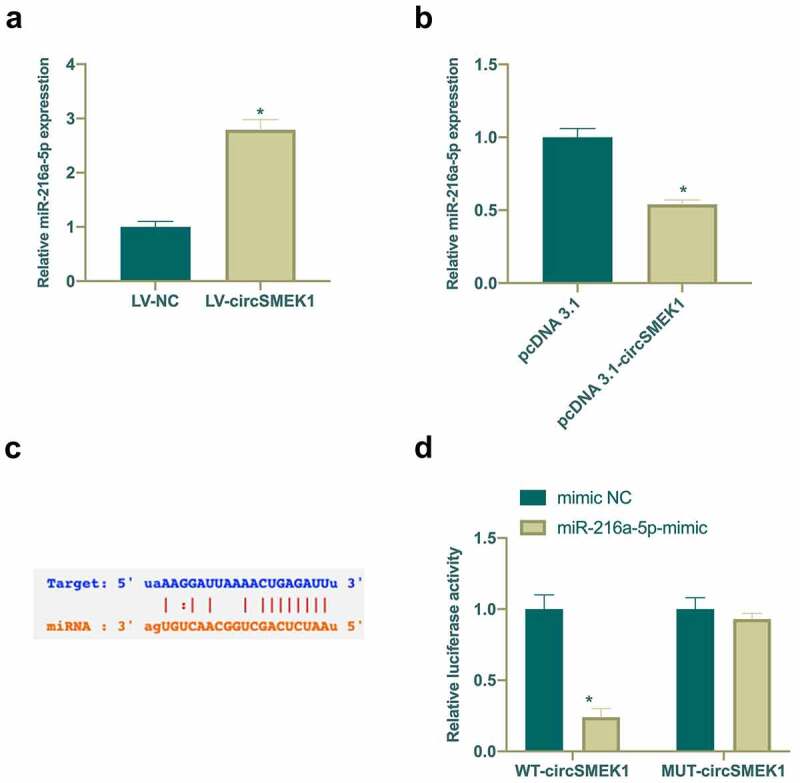
circSMEK1 competitively binds to miR-216a-5p

Next, the potential relationship between circSMEK1 and miR-216a-5p was checked, finding that after silencing or overexpressing circSMEK1, miR-216a-5p levels in NP model were elevated and reduced separately (Figure 4(a,b)). Therefore, it was speculated that circSMEK1 could target miR-216a-5p. Through the search on http://starbase.sysu.edu.cn/ found that circSMEK1 and miR-216a-5p have potential binding sites (Figure 4(c)). Later, DLR experiment was applied for further verification of their targeting relationship. WT circSMEK1 apparently reduced the luciferase activity of miR-216a-5p mimic group, while MUT circSMEK1 visually had no effects on it (Figure 4(d)). This indicated that circSMEK1 targets miR-216a-5p expression.

A. RT-qPCR detection of miR-216a-5p expression in LV-NC and LV-circSMEK1 groups; B. RT-qPCR detection of miR-216a-5p expression in pcDNA3.1 and pcDNA3.1-circSMEK1 groups; C. Queried the potential binding sites of circSMEK1 and miR-216a-5p through http://starbase.sysu.edu.cn/; D; Detection of targeting relationship between circSMEK1 and miR-216a-5p via DLR experiment; Data presentation detailed as mean ± SD (A, n = 9; B and D, n = 3), * vs LV-NC, pcDNA3.1, or mimic NC groups, *P* < 0.05.

### 3.5. TXNIP is a target gene of miR-216a-5p

**Figure 5.**
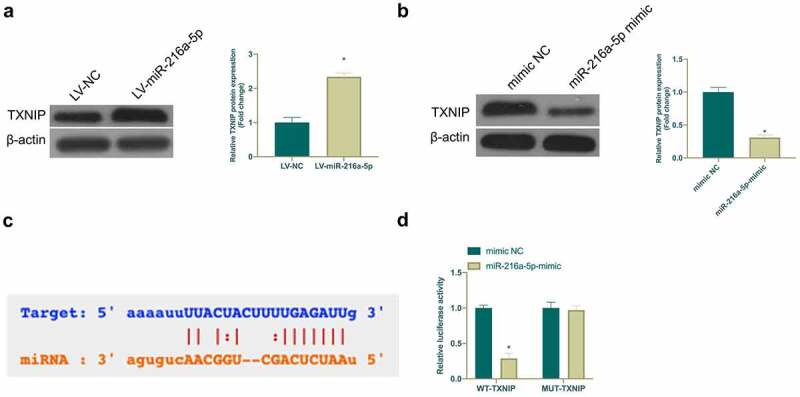
TXNIP is targeted via miR-216a-5p

MiRNA usually binds to mRNA 3ʹUTR to modulate gene expression [24]. Next, the target gene of miR-216a-5p was exploited. Based on previous studies, TXNIP is highly expressed in NP or nerve damage and plays an essential role. In this work, it was found that TXNIP expression was elevated in CCI rats and LPS-treated microglia, while overexpressing or silencing miR-216a-5p inhibited or promoted TXNIP expression, respectively (Figure 5(a,b)). It was speculated that TXNIP may be a target gene of miR-216a-5p. Through searching on http://starbase.sysu.edu.cn/ found that TXNIP and miR-216a-5p have potential-binding sites (Figure 5(c)). Additionally, DLR experiment was given further check that WT TXNIP reduced the luciferase activity of miR-216a-5p mimic group, but MUT TXNIP had no clear effect on it (Figure 5(d)). These findings indicated that TXNIP is a potential target gene of miR-216a-5p.

A. Western blot detection of TXNIP expression of LV-NC and LV-miR-216a-5p groups; B. Western blot detection of TXNIP expression in microglia of mimic NC and miR-216a-5p-mimic groups; C. Checked the potential-binding sites of TXNIP and miR-216a-5p via website http://starbase.sysu.edu.cn/; D; DLR experiment detection of the targeting relationship of TXNIP and miR-216a-5p; Data presentation detailed as mean ± SD (A, n = 9; B and D, n = 3); * vs LV-NC or mimic NC groups, *P* < 0.05.

### 3.6. CircSMEK1 promotes NP through the miR-216a-5p/TXNIP axis

**Figure 6.**
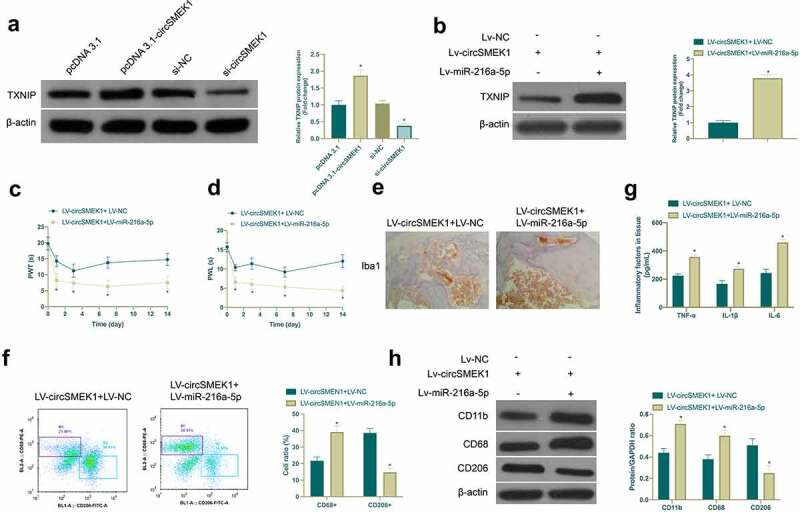
circSMEK1 promotes NP through the miR-216a-5p/TXNIP axis

For exploring whether miR-216a-5p/TXNIP axis was involved in the process of circSMEK1 regulating NP, the effect of circSMEK1 on TXNIP protein expression was first examined (Figure 6(a)), assuring that depression or overexpression of circSMEK1 declined or elevated TXNIP in microglia. Then, LV-circSMEK1 + LV-miR-216a-5p lentiviral vector was received with co-injection into CCI rats. LV-circSMEK1 signally reduced TXNIP expression, while simultaneous injection of LV-miR-216a-5p effectively restored TXNIP expression (Figure 6(b)). Additionally, the beneficial effects posed by LV-circSMEK1 on CCI rats were visually reversed by simultaneous injection of LV-miR-216a-5p, mainly manifested as aggravated mechanical allodynia and thermal hyperalgesia (Figure 6(c,d)), Iba1 positive cells (Figure 6(e)), CD68+ cells (Figure 6(f)) and TNF-α, IL-1β and IL-6 levels (Figure 6(g)), along with elevated CD11b and CD68 expression but reduced CD206 expression and CD206+ cells (Figure 6(h,f)). Briefly, circSMEK1 accelerates NP progression through the miR-216a-5p/TXNIP axis.

In LV-circSMEK1+ LV-NC and LV-circSMEK1+ LV-miR-216a-5p groups, A. Western blot detection of the effect of knockdown or overexpression of circSMEK1 on TXNIP expression in microglia; B. Western blot detection of TXNIP expression in rat spinal cord tissues; C and D. Mechanical pain threshold and withdrawal latency to thermal stimulation in plantar test; E. Immunohistochemistry detection of Iba1 expression in spinal cord tissue; F. Flow cytometry detection of the ratio of CD68+ and CD206+ cells in rat spinal cord; G. ELISA detection of TNF-α, IL-1β and IL-6 levels in spinal cord tissue; H. Western blot detection of CD11b, CD68 and CD206 expressions in spinal cord tissue; Data presentation detailed as mean ± SD (n = 9); * vs LV-circSMEK1 + LV-NC group, *P* < 0.05.

## 4. Discussion

In recent years, despite advances in clinically treating NP, many patients still suffer from chronic pain and psychological distress [25]. NP’s underlying pathogenesis is unclear. This work found that a new type of circRNA SMEK1 is available to promote NP in CCI rats and LPS-treated microglia, including pain value, inflammation, and pro-inflammatory phenotype transformation in microglia. Further mechanism studies have revealed that circSMEK1 promotes NP development via competitively binding miR-216a-5p to mediate TXNIP expression.

Increasing studies have supported that circRNA is a key regulator in NP development. In serrated animal NP model, it has been found that circ_0005075, circZNF609, circZRANB1, cirS-7, circHIPK3, etc. have vital relationships with NP development [26–29]. It was in this work found that overexpressed circSMEK1 promotes NP development and spinal cord inflammation in CCI rats. When NP occurs, the phenotypes of microglia usually change, with an increase in pro-inflammatory microglia [30,31]. In this work, it was manifested that overexpressed circSMEK1 increased CD68 expression in CCI rats and LPS-treated microglia while inhibited CD206 expression. That is probably why circSMEK1 regulates neural pain. It is worth noting that a growing number of studies have manifested that central sensitization is driven by peripheral and central nervous system neuroinflammation. Activation of microglia and transformation into pro-inflammatory types, result in the release of pro-inflammatory cytokines and chemokines, which provides sufficient support for central nervous system hyperalgesia [32]. In addition, microglia activation [33] is often found in sawtooth animal models of neuropathic pain producing strong mechanical stimuli. Mechanical pain in sawtooth animals can be effectively improved by changing microglia phenotype [34]. Recently, Zhang S B *et al*. found that circANKS1A promotes NP model hypersensitivity in serrated animals. Moreover, Wei M *et al*. found that circZRANB1 reduces mechanical pain in NP model in serrated animals [35]. The results of this study affirmed that circSMEK1 modulated spinal cord inflammation by influencing microglia M1/M2 polarization, thus affecting NP mechanical pain and pain allergy, which further demonstrated the potential of circRNA in controlling central nervous microglia activation. CircRNA needs to sponge downstream miRNAs and regulate corresponding target genes so as to function [36,37]. Further mechanism studies have supported that circSMEK1 acts as a miR-216a-5p sponge to mediate TXNIP expression to promote NP. In reference to the latest research, miR-216a-5p reduces NP, inflammatory factor expression and microglia infiltration in rats via targeting KDM3A and inactivating the wnt/β-catenin signaling pathway. Additionally, Wei L *et al*. found that miR-216a-5p from mesenchymal stem cell exosomes is available to repair spinal cord injury via changing M1/M2 polarization in microglia, suggesting that miR-216a-5p has great potential in improving NP. It was in this work assured that overexpressed miR-216a-5p inhibited nerve pain in CCI rats, reduced spinal cord inflammation and microglia activation, and transformed microglia to be anti-inflammatory. Additionally, the same findings were achieved through *in vitro* experiments. TXNIP is an endogenous negative regulator belonging to the α-arrestin protein family in TRX system, and widely expressed in almost all normal tissue cells [38]. It usually binds to the NLRP3 inflammasome to affect apoptosis, oxidative phosphorylation and inflammation [39]. Previous studies have revealed that miR-23a/CXCR4 modulates NP via targeting the TXNIP/NLRP3 inflamed body axis [40]. Recently, it has also been found that NF-κB-dependent transcriptional regulation promotes CCI-induced NP through the miR-183/TXNIP/NLRP3 axis [41]. This study found that circSMEK1 and miR-216a-5p regulated NP mainly via relying on targeted regulation of TXNIP expression. This further reveals the vital role of TXNIP playing in nerve injury.

## 5. Conclusion

To sum up, this work supports that circSMEK1 competitively binds miR-216a-5p to target and regulate TXNIP expression to promote NP, spinal cord inflammation and CCI-caused microglia activation. These data further reveal NP’s underlying pathogenesis and provide possible therapeutic targets.
